# Intratracheal Administration of Itraconazole-Loaded Hyaluronated Glycerosomes as a Promising Nanoplatform for the Treatment of Lung Cancer: Formulation, Physiochemical, and In Vivo Distribution

**DOI:** 10.3390/pharmaceutics16111432

**Published:** 2024-11-10

**Authors:** Sultan Aati, Hanan O. Farouk, Marwa H. Elkarmalawy, Hanan Y. Aati, Nahla Sameh Tolba, Hossam M. Hassan, Mostafa E. Rateb, Doaa S. Hamad

**Affiliations:** 1 Dental Health Department, College of Applied Medical Sciences, King Saud University, Riyadh 11421, Saudi Arabia; sati@ksu.edu.sa; 2 Department of Pharmaceutics, Faculty of Pharmacy, Nahda University, Beni-Suef 62521, Egypt; hanan.osman@nub.edu.eg; 3 Department of Pharmaceutics and Drug Manufacturing, Faculty of Pharmacy, Modern University for Technology and Information, Cairo 11571, Egypt; marwa.hesham@pharm.mti.edu.eg; 4 Department of Pharmacognosy, College of Pharmacy, King Saud University, Riyadh 11495, Saudi Arabia; 5 Department of Pharmaceutics, Faculty of Pharmacy, Sadat City University, Sadat City 32897, Egypt; nahla.sameh@fop.usc.edu.eg; 6 Department of Pharmacognosy, Faculty of Pharmacy, Beni-Suef University, Beni-Suef 62521, Egypt; 7 School of Computing, Engineering & Physical Sciences, University of the West of Scotland, Paisley PA1 2BE, UK; mostafa.rateb@uws.ac.uk; 8 Department of Pharmaceutics and Pharmaceutical Technology, Faculty of Pharmacy, Nile Valley University, Fayoum 63518, Egypt; doaa.qarni@nv.edu.eg

**Keywords:** itraconazole, hyaluronated glycerosomes, A549 cell line, tumor targeting, intratracheal, in vivo biodistribution

## Abstract

Background: Itraconazole (ITZ) is an antiangiogenic agent recognized as a potent suppressor of endothelial cell growth that suppresses angiogenesis. Nevertheless, its exploitation is significantly restricted by its low bioavailability and systematic side effects. The objective of this study was to utilize glycerosomes (GLY), glycerol-developed vesicles, as innovative nanovesicles for successful ITZ pulmonary drug delivery. Methods: The glycerosomes were functionalized with hyaluronic acid (HA-GLY) to potentiate the anticancer efficacy of ITZ and extend its local bio-fate. ITZ-HA-GLY were fabricated using soybean phosphatidylcholine, tween 80, HA, and sonication time via a thin-film hydration approach according to a 24 full factorial design. The impact of formulation parameters on ITZ-HA-GLY physicochemical properties, as well as the optimal formulation option, was evaluated using Design-Expert^®^. Sulphorhodamine-B (SRB) colorimetric cytotoxicity assay of the optimized ITZ-HA-GLY versus ITZ suspension was explored in the human A549 cell line. The in vivo pharmacokinetics and bio-distribution examined subsequent to intratracheal administrations of ITZ suspension, and ITZ-HA-GLY were scrutinized in rats. Results: The optimized ITZ-HA-GLY unveiled vesicles of size 210.23 ± 6.43 nm, zeta potential of 41.06 ± 2.62 mV, and entrapment efficiency of 73.65 ± 1.76%. Additionally, ITZ-HA-GLY manifested a far lower IC50 of 13.03 ± 0.2 µg/mL on the A549 cell line than that of ITZ suspension (28.14 ± 1.6 µg/mL). Additionally, the biodistribution analysis revealed a higher concentration of ITZ-HA-GLY within the lung tissues by 3.64-fold as compared to ITZ suspension. Furthermore, the mean resistance time of ITZ-HA-GLY declined more slowly with 14 h as compared to ITZ suspension, confirming the accumulation of ITZ inside the lungs and their promising usage as a target for the treatment of lung disease. Conclusions: These data indicate that the improved ITZ-HA-GLY demonstrates significant promise and represents an exciting prospect in intratracheal delivery systems for lung cancer treatment, meriting further investigation.

## 1. Introduction

Among all cancers, lung cancer is quite frequent and stands high in regard to cancer-related fatalities worldwide. Lung cancer has a greater mortality rate (around 15%) than prostate, breast, or colon cancer because of the difficulty in diagnosing the disease and the high risk of metastases [1,2]. The symptoms can be highly unpredictable, attributable to the great variety of the disease and the fact that it might manifest in various parts of the lungs. The two principal types of lung cancer are small cell lung cancer (SCLC) and non-small cell lung cancer (NSCLC), the former of which is more prevalent [2]. The main methods for treating lung cancer include radiation and chemotherapy. The problems with both methods include drug resistance, dosage-dependent toxicity, poor selectivity, and insufficient drug concentrations throughout the lungs due to increased interstitial pressure [3]. Thus, innovative treatments with reduced occurrence of adverse reactions and increased effectiveness in treatment are required [4]. Producing anti-cancer drugs is costly and time-consuming, but there are methods of overcoming this by finding new uses for existing medications that have already been tested for human toxicity or by creating anti-tumor therapies that have already been approved for chemotherapy by the FDA [3].

Itraconazole (ITZ), a well-tolerated antifungal medication, is classified as a triazole molecule. Recent evidence from preclinical and clinical studies has confirmed the effectiveness of ITZ in treating many types of cancer. This supports the idea of repurposing ITZ as a viable chemotherapy drug [5]. One of the main ways that ITZ works is by blocking the mechanism for hedgehog signaling [6], impeding the growth of tumors and new blood vessels while enhancing cell death and autophagy [7]. ITZ further impairs the P-glycoprotein efflux pump, hence counteracting chemoresistance [6,8]. The therapeutic effects of ITZ have been accepted by current clinical trials evaluating a diversity of cancer types, including basal cell carcinoma, triple-negative breast cancer, ovarian cancer, and prostate cancer [9].

The intratracheal administration method used for treating respiratory diseases is notable for being painless, highly effective, and non-invasive. This approach allows drugs to target the organ level directly, reducing drug loss during circulation by avoiding first-pass metabolism in the liver. As a result, it maximizes localized concentration and efficacy while reducing systemic distribution and potential adverse effects, ultimately enhancing patient adherence to treatment. Consequently, intratracheal drug delivery represents a significant advancement in lung cancer therapy [5]. Intratracheal delivery of nanovesicles, such as liposomes, is an encouraging non-invasive approach for treating respiratory diseases. By regulating the size of these nanovesicles, they can effectively reach the alveolar region as they have Enhanced Permeability and Retention (EPR) that allows them to have uniform distribution, enhanced deposition, and improved delivery efficiency deep within the lung cells [10,11]. However, the intricate biological barriers in the lungs, including limited permeability of the lung epithelium, enzymatic degradation, and mucociliary clearance, pose significant challenges to the inhalation delivery of liposomes [12]. To tackle these challenges, modifying the surface properties of inhalable liposomes is a viable approach. This adjustment not only influences their permeability through mucus and the epithelium but also impacts their retention and penetration abilities within the respiratory tract [13,14].

Glycerosomes (GLY) represent a new concept that evolved from liposomes. These innovative patented phospholipid vesicles have a submicron size range of 100–1000 nm. Incorporating phospholipids, glycerol, and water, they function as innovative vesicular carriers for trans-mucosal drug delivery. Glycerosomes are created using a variety of phospholipids and contain a significant amount of glycerol (20–40%, *v*/*v*) [15]. They are versatile vesicular carriers, and the glycerol portion alters the fluidity of the vesicle membrane. They are composed of various substances, including cholesterol, which contributes to the stability of the lipid bilayer [13,14]. However, the potential of these vehicles is limited by their physical instability and rapid metabolism, restricting their applicability.

Polymeric glycerosomes are paradigms derived from glycerosomes. These vesicles create an internal mesh of polymers, enhancing the in vivo characteristics of the system. Notable features of polymeric glycerosomes include improved biocompatibility, better drug deposition at target sites, avoidance of first-pass metabolism, and effective delivery of contents to specific locations in the lungs, all while being safe and non-toxic. Additionally, these formulations can be easily transformed into spray form, facilitating pulmonary drug delivery [16].

An anionic polysaccharide, hyaluronic acid (HA) has a variable amount of disaccharide units that repeat, specifically d-glucuronic acid and N-acetyl-d-glucosamine. This naturally occurring polymer is widely present in the human body, particularly in connective tissues, eye structures, intestines, and lung tissues [17]. It plays an essential function in the function of various inflammatory mediators and enhances the transit rate of mucus in the airways due to the polymer’s capability to cling to mucus [18,19]. Moreover, formulations for bilayer vesicles intended for pulmonary delivery frequently incorporate it due to its natural origin, ease of degradation, elastic-viscous properties, and stabilizing functions [20]. Interestingly, a multi-functional transmembrane glycoprotein, CD44 is known for its dual roles as a hyaluronic acid (HA) receptor and a homing receptor for human cells. [21]. It is a glycoprotein located on the cell surface that binds hyaluronic acid (HA) and is involved in various normal and abnormal cellular functions, such as cell migration, wound healing, lymphocyte homing, proliferation, invasion, and metastasis [22]. In contrast to normal cells, numerous cancer cells, such as those from lung, breast, pancreatic, gastric, and colon tumors, have heightened expression of CD44, representative its potential as a potential target for cancer therapy [23].

In light of the aforementioned information, It was expected that intratracheal administration of ITZ enclosed in GLY with HA surface functionalization would significantly enhance the therapeutic index of ITZ through improved physicochemical qualities, targeted drug deposition, and enhanced mucus barrier penetration. Consequently, in this research, we intended to develop a combinatorial system utilizing ITZ-HA-GLY for administration via the intratracheal (i.t) route to facilitate effective pulmonary delivery of ITZ. A concentrated and stable ITZ-HA-GLY formulation with superior physicochemical properties and appropriate nanovesicular size was established by full factorial design. The physicochemical properties of the designed ITZ-HA-GLY were evaluated to ascertain its suitability for pulmonary administration. We further examined the anticancer effects of an aqueous ITZ suspension and ITZ-HA-GLY on the A549 lung cancer cell line by evaluating cell viability. Finally, the bio-distribution of ITZ from ITZ -HA-GLY nanosuspension was compared with aqueous ITZ suspension after Intratracheal given in rats.

## 2. Materials and Methods

### 2.1. Materials

ITZ kindly supplied by Memphis Company (Cairo, Egypt), whereas soybean phosphatidylcholine, Tween 80 (polyoxyethylene sorbitan monooleate), hyaluronic acid sodium salt from Streptococcus equi (Sodium hyaluronate, with a molar mass LMw ≈ 8000–15,000 Da, acetonitrile, potassium dihydrogen orthophosphate, dimethyl sulfoxide (DMSO), sulforhodamine dye, and trichloroacetic acid were provided by Sigma-Aldrich (St. Louis, MO, USA). Polyoxyethylene sorbitan monooleate, glacial acetic acid, and glycerol were acquired from Al Naser Company (Cairo, Egypt). A549 cell lines and HSF-1 cell lines were supplies by (ATCC, Alexandria, MN, USA). Dialysis bags with a molecular weight cutoff of 12,000 Da were obtained from SERVA Electrophoresis GmbH (Heidelberg, Germany). All utilized components were of analytical grade.

### 2.2. Fabrication of ITZ-HA-GLY

The thin-film hydration approach was utilized to produce the ITZ-HA-GLY. The first step was to dissolve soybean phosphatidylcholine, tween 80, and ITZ in 10 mL of chloroform. The organic solution was then put into a flask with a round bottom and heated to 60 °C. An extremely thin layer developed on the inside of the flask as a result of the chloroform being vaporized in a vacuum employing a rotary evaporator (Heidolph Laborota 4000 Series, Heizbad, Germany) running at 90 rpm. Subsequently, the film had become hydrated by adding 10 mL of dispersion containing HA in a glycerol/water mix (1/1 *v*/*v*), resulting in the formation of ITZ-HA-GLY. Bath-sonication was performed on the prepared ITZ-HA-GLY using a Tianjin Automatic Science Instrument Ltd. instrument from Nanyang, China [24]. Afterward, overnight at 4 °C, the vesicle dispersion was allowed to develop prior to undertaking any additional investigations.

### 2.3. Experimental Design

A 2^4^-factorial design examined the impact of factors on ITZ-HA-GLY characteristics. The Design-Expert^®^ program (Version 10, Stat-Ease Inc., Minneapolis, MN, USA) was used for this purpose. Variables that were manipulated independently in the experiment were concentrations of soybean phosphatidylcholine (X_1_), HA (X_2_), tween 80 (X_3_), and sonication time (X_4_). Entrapment efficiency (Y1), vesicle size (Y2), and zeta potential (Y3) were the dependent variables that were examined.

### 2.4. ITZ-HA-GLY Characterization and Optimization

#### 2.4.1. Analysis of Entrapment Efficiency (EE)

The EE of ITZ-HA-GLY was established by rotating corresponding dispersions at 14,000 rpm for 2 h at 4 °C (Sigma Laborzentrifugen D-37520, Osterode-am-Harz, Germany) [25]. Each time, the translucent supernatant was separated from precipitated ITZ-HA-GLY and filtered through a 0.45 nm nylon syringe filter. Drug non-entrapped was then tested at 264 nm using a UV spectrophotometer (Shimadzu UV-1800, Tokyo, Japan). A range of 5-13 μg/mL was covered in a calibration curve that was produced in PBS with a pH of 7.4 before the analysis. (R^2^, 0.9926). EE of ITZ remained determined utilizing the subsequent Formula (1):

EE% = (Total amount of ITZ − free amount ITZ)/(Total amount of ITZ) × 100(1)

#### 2.4.2. Analysis of Vesicle Size, Polydispersity Index, and Zeta Potential of ITZ-HA-GLY

The polydispersity index (PDI), zeta potential (ZP), and average diameter for each formulations was assessed using photon correlation spectroscopy with a Zetasizer 2000 (Malvern Instruments Ltd., Malvern, UK). 1 mL of the ITZ-HA-GLY was diluted with 10 mL of distilled water and subsequently measured. Mean values ± SD obtained were recorded, and the triplicate measurements were implemented, with the temperature at 25 ± 2 degrees Celsius and the angle equal to 90 degrees with respect to the beam of incident [26].

#### 2.4.3. Optimization of ITZ-HA-GLY

The desirability function was employed to determine the optimal ITZ-HA-GLY dispersion using the Design-Expert^®^ program. The optimization method was developed to choose a formula that has the highest EE and ZP while also having the smallest vesicle size. The choice was made for the solution with an attractiveness value that was nearly one. To emphasize the validity of the model, the chosen formulation has been developed, assessed, and compared to the predicted results.

### 2.5. Characterization of the Optimized Formulation

#### 2.5.1. Transmission Electron Microscopy (TEM)

A high-sensitivity transmission electron microscope known as JEM-1400 was used to examine the improved ITZ-HA-GLY’s morphology (JEOL Ltd., Tokyo, Japan). The sample was positioned on a copper-gold carbon matrix until fully dried, then grid-inserted into the microscope, and images were captured at varying magnification levels [27].

#### 2.5.2. In Vitro Drug Release Study

The properties of ITZ release from ITZ-HA-GLY were investigated in vitro and compared to ITZ suspension using dialysis bag diffusion. The release investigation was performed at two distinct pH levels: 7.4, representing normal physiological pH, and 5.5, indicative of endosomal pH. which contained a 0.5% solution of sodium lauryl sulfate. The purpose of adding sodium lauryl sulfate was to enhance the solubility of ITZ. The investigation was conducted for a duration of 24 h [28]. The drug release process was started by placing the dialysis tube in 200 mL of release media on a magnetic stirrer at a temperature of 37 ± 5 °C and a speed of 100 rpm. At predetermined time intervals, samples of 5 mL were taken, and the concentration of the drug was measured using a UV/Visible spectrophotometer (Jasco V530, Tokyo, Japan) at a wavelength of 264 nm. To guarantee that the sink condition was sustained for 24 h, each sample was expelled from the medium and substituted with a new one.

The data on the release of the drug were analyzed empolying DDSolver, an Excel plugin, and the resulting data were fitted to various kinetic models. The DDSolver software was employed to reduce computation time, minimize calculation errors, and ascertain the accurate release data [29]. Following the obtaining of the release data, the data were imported into the DDSolver tool to identify the six most significant and prevalent criteria. coefficient of determination (Rsqr, R^2^), Akaike Information Criterion (AIC), Model Selection Criterion (MSC) and n (release exponent). First order, Higuchi, Korsmeyer–Peppas, Hopfenberg, and PeppasSahlin were evaluated using the values that had the highest R^2^ and MSC values, while the values with the lowest AIC values were picked. Finally, release differences of prepared ITZ-HA-GLY at different pHs (5.5 and 7.4) were evaluated by DDSolver [30].

#### 2.5.3. In Vivo Study

##### Animals

Animals used in this study were 84 male Albino Westar rats weighing 180–200 g, purchased from the Faculty of Pharmacy, Nahda University’s (Beni Suef, Egypt). The rats were housed in the animal care facility and provided with food and water at desire and under controlled lighting conditions. All the techniques for drug administration and blood and tissue collection during the experiment were assented by the Animal Ethics Committee of the Faculty of Pharmacy, Nahda University [31].

##### ITZ Biodistribution

To quantify the amount of ITZ in the different tissues after intratracheal administration of both ITZ-HA-GLY and free ITZ, 84 male Albino Wistar rats were separated into two groups at unplanned (n = 6/group) and were intratracheally administered with a dose of 10 mg/kg of ITZ (drug suspension and ITZ-HA-GLY). A single dose of ITZ-HA-GLY, equivalent to the drug dose, was administered to each rat in the first group. At the same time, the second group was given a suspension (2 mg/mL) of free ITZ in phosphate buffer (pH 6.8).

##### Plasma and Organs

At 0.5, 1, 2, 3, 4, 6, and 24 h after intratracheal administration of ITZ-HA-GLY and free ITZ (n = 6/group for each treatment/time) were anesthetized (ketamine 60 mg/kg and xylazine 7.5 mg/kg) then slaughtered by cervical dislocation. The blood was directly centrifuged at adjusted conditions of 6000 rpm and 4 °C for a duration of 5 min after being assembled via cardiac puncture to separate the plasma, then the plasma was kept overnight at −70 °C prior to LC-MS/MS analysis. After the animal was euthanized, the liver, spleen, lungs, and kidneys were separated and rinsed with cold saline, blotted dry on a filter paper, and their weight was recorded. The separated tissue samples were then homogenized using an Ul-tra-Turrax^®^ T25 homogenizer (Co. KG, Staufen, Germany) with a 30:70 (*v*/*v*) water–acetonitrile mixture. The resulting supernatant was collected by centrifugation at 6000 rpm and 4 °C for 30 min and was stored at −70 °C until analysis [32,33].

##### Chromatographic Conditions

The concentrations of ITZ from ITZ-HA-GLY and ITZ suspension in the separated plasma and homogenized organs were analyzed quantitatively using the LC-MS/MS detection method reported and validated by Py-Daniel et al. [34]. The analysis was executed using an LC-MS/MS system (Foster City, CA, USA). attached to a Triple Quad API-4000 mass spectrometer (PE Sciex, canada) provided with a Turbo Ion Spray™ interface at 350 °C. The chromatographic separation was carried out on the analytical column of Hypersil C18 (250 mm × 3.9 mm, 5 μm). An elution program was used to pump the mobile phase containing 80% acetonitrile and 20% of 0.01 M potassium dihydrogen orthophosphate at a flow rate of 1 mL/min. ITZ was identified at the wavelength of 264 nm.

##### Analysis of ITZ Plasma and Tissue Samples

To quantify ITZ in plasma, 1 mL of plasma was mixed with 1 mL of acetonitrile and vortexed for 1 min, followed by another minute of vortexing. Next, the top organic layer was extracted using centrifugation at 5000 rpm and 4 °C for 10 min using a cooling centrifuge (model 2–16 PK; Sigma). The solvent was evaporated completely by placing the test tube under a stream of nitrogen at 50 °C for 45 min. The remainder was then re-constituted in 80 µL of the mobile phase. The sample was vortexed for 5 min, centrifuged at 3000 rpm for 10 min to separate the supernatant, and 20 µL of this translucent supernatant was injected into the column for detection. For detecting ITZ in tissues, the spleen, lung, and kidney were individually homogenized and extracted using the same method as for plasma (the liver was handled separately due to its large mass, with only 400 mg used). Prior to plasma and tissue extraction, 60 µL of paracetamol IS (from a 100 ng/mL stock solution) was added to the tubes [33].

#### 2.5.4. Cytotoxicity Evaluation Utilizing Sulforhodamine B Colorimetric Assay (SRB)

##### Cell Culture

Human skin fibroblast cell lines (HSF-1) and lung cancer cell line A549 were obtained from (ATCC, Alexandria, MN, USA). and kept at the National Cancer Institute, Cairo, Egypt, through serial sub-culturing. Samples were produced by dissolving 1:1 stock solution and stored at −20 °C in DMSO. SRB dye was dissolved in 1% glacial acetic acid. A 10% solution was utilized for protein precipitation, and a 50% stock solution of trichloroacetic acid (TCAa) was produced. The SRB dye was dissolved in 10 mM of Tris base (pH 10.5). To make it, we dissolved 121.1 g of tris base in 1000 mL of pure water and then corrected the pH with 2 M hydrochloric acid.

##### Anticancer Activity

The cytotoxicity was assessed using SRB colorimetric assay following the method reported by Skehan, 1990. a bright pink aminoxanthrene dye SRB has two sulphonic groups. It is a protein stain that attaches to the amino groups of intracellular proteins in mildly acidic circumstances, serving as a sensitive indicator of cellular protein levels [35].

In 96-well microtiter plates, A549 cell lines were seeded with 200 µL of fresh media at an initial concentration of 4 × 103 cell/well and allowed to adhere to the plates for 24 h. ITZ suspension and ITZ-HA-GLY were added at several concentrations (62.50 to 500.00 µg/mL). For each drug concentration (ITZ suspension and ITZ-HA-GLY) three wells were utilized. Subsequently, the plates were incubated for 48 h, after which the cells were fixed using 10 μL of cold trichloroacetic acid at a final concentration of 10% for 1 h at 4 °C. The plates were rinsed with distilled water utilizing an automatic washer (Tecan, Crailsheim, Germany). 

After that, they were stained with 50 μL of 0.2% SRB mixed in 1% acetic acid and left to sit in the dark at room temperature for 30 min. The plates were subsequently air dried after being rinsed with 1% acetic acid. Finally, the dye was solubilized with 200 μL/well of 10 M tris base (pH 10.5), and the optical density (O.D.) of each well was measured spectrophotometrically at 570 nm using an ELISA microplate reader (Sunrise Tecan reader, Essen, Germany). Each drug concentration’s mean value was computed after automatically subtracting the mean background absorbance. The cell survival % was determined as follows, in Formula (2):

(2) Surviving fraction=O.D.(treated cells)/O.D.(control cells).

In addition, IC_50_ values, which are the concentrations of the ITZ that must be present in order to inhibit cell growth by 50%, were computed using Prism version 5 (San Diego, CA, USA) [36].

##### Cytotoxicity on Human Normal Cell Line (HSF-1)

The identical technique was executed for A549 cell lines to evaluate the cytotoxic effects of ITZ suspension and ITZ-HA-GLY on a normal cell line. The formulations were evaluated within the identical dosage range utilized for the A549 cell lines.

### 2.6. Statistical Analysis

One-way analysis of variance (ANOVA) was implemented to conduct statistical analyses followed by Tukey’s post-hoc test using Design-Expert^®^ program (Version 10) and GraphPad (Prism 10.4.0, USA). A *p*-value that were less than 0.05 were regarded as statistically significant.Utilizing PK-Solver, a complimentary, menu-driven Microsoft Excel add-in(version 2019), non-compartmental pharmacokinetic analysis was implemented to ascertain all pharmacokinetic parameters and DD-Solver for the kinetic release model [37].

## 3. Results

### 3.1. Fabrication of ITZ-HA-GLY

ITZ-HA-GLY was successfully developed by adopting Manca et al.’s method [13]. HA-GLY vesicles were produced by amalgamating glycerosomes with biocompatible polymers through a single-step, environmentally sustainable technique: Soybean phosphatidylcholine, glycerol, water, and sodium hyaluronate. ITZ (10 mg/mL) was successfully incorporated in the vesicles by direct hydration of ITZ and Soybean phosphatidylcholine with a glycerol/water solution (50% *v*/*v*) and HA to obtain HA-glycerosomes. The physicochemical properties of HA-glycerosomes and their in vitro efficacy are always more promising than those of drug suspension.

### 3.2. Experimental Design and Optimization

Utilizing the Design-Expert^®^ Software(Version 10, Stat-Ease Inc., Minneapolis, MN, USA), statistical analysis was implemented, and the design used was a 2^4^ full factorial design. Table 1 exhibits independent factors at both low and high levels, as well as the dependent variable. Preliminary testing and feasibility of developed ITZ-HA-GLY at the tested values were used to determine the levels of each factor. Specifically, a two-factor interaction (2FI) model was chosen. A sufficient signal-to-noise ratio was assessed to ensure the model’s usability in navigating the design space [38]. All assessed responses had a ratio exceeding 4, which is considered appropriate. Furthermore, it is desirable for the values of adjusted and predicted R^2^ to align closely in order to achieve a realistic harmony [39]. The composition of the selected formulations is detailed in Table 2. Table 2 outlines the experimental runs and the responses that were measured. Table 3 also shows the analysis of variance for all factors in a modeled response that has been computed after being measured, including effects, degrees of freedom, F-ratio, and *p*-value.

#### 3.2.1. Analysis of Entrapment Efficiency (EE)

**Figure 1:**
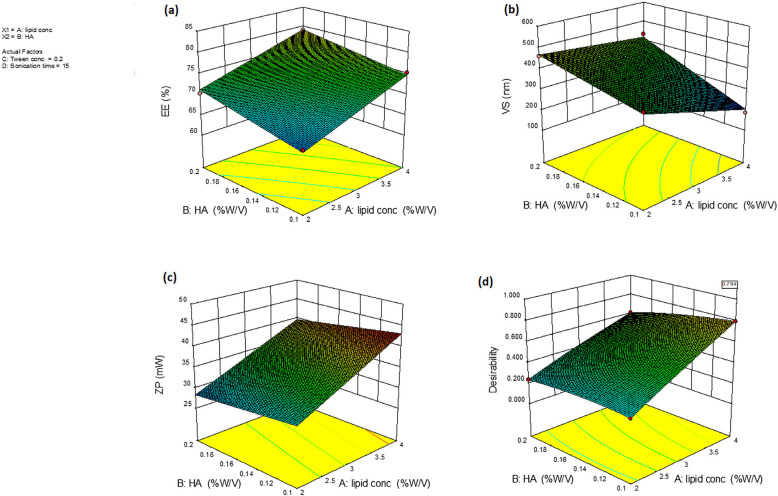
Response surface plot for the effect of concentration of lipid (soybean phosphatidylcholine) (X1), HA (X2) at the middle levels of the 3rd and 4th variables (tween 80 concentration and sonication time) on (**a**) EE%, (**b**) VS, (**c**) ZP and (**d**) desirability of the developed ITZ-HA-GLY.

The ITZ-HA-GLY formulations successfully entrapped a significant quantity of ITZ. The percent of ITZ retained by these formulations varied between 63.46 ± 0.65% and 83.97 ± 1.23%. The high EE is due to the presence of multilayered smooth vesicles, which allowed for a higher volume of entrapped; these data can be seen in Table 2. Figure 1a depicts the response surface plot that shows how two independent factors together affect the EE of ITZ-HA-GLY at the middle level of the third variable. According to Table 3, the F-value of 20.27, which was statistically significant (*p* < 0.002), was obtained by best-fitting the EE data to the quadratic model. The regression equation (in coded values) that contributes to the impact of the four independent variables on the EE (Y1) is represented by Formula (3):

EE = 65.90 + 4.03X_1_ + 2.40X_2_ + 2.48X_3_
*−* 5.04X_4_
*−* 0.22X_1_X_2_ + 0.66 X_1_X_3_ + 0.47X_1_X_4_ + 0.54X_2_X_3_ + 0.50X_2_X_4_ + 0.64X_3_X_4_ (3)

Due to the amphiphilic nature of both soybean phosphatidylcholine and tween 80, the positive coefficient of the concentration of soybean phosphatidylcholine and tween 80 shows that, as the concentration of these components increases, the EE of ITZ also increases. The lipophilicity of ITZ allows for its easy incorporation into the soybean phosphatidylcholine bilayer of vesicles, resulting in a greater soybean phosphatidylcholine surface area existing for entrapment. Additionally, the presence of a larger amount of vesicles provides more opportunities for ITZ to be entrapped into the outer soybean phosphatidylcholine phase, thereby enhancing the EE [40]. Additionally, the positive influence of tween 80 concentration on ITZ can be attributed to the tween 80’s ability to provide additional space for retaining more drug. This can result in the development of a monomolecular tween 80 layer that helps stabilize the interface of the vesicles [41].

The existence of HA has a major impact on EE, which can be attributed to two mechanisms. Firstly, the ionic interaction between 80, the positive charge of ITZ, and the negatively charged carboxyl group of HA resulted in the entrapping of a greater quantity of drug in the hyaluronated core of the prepared glycersome, thereby reducing drug leakage [42]. Secondly, the presence of HA increases the viscosity of the inner core of the vesicles and the surrounding aqueous environment, resulting in a reduction in drug discharge from the vesicles [43].

It was obvious that the EE values of ITZ-HA-GLY were significantly influenced by the sonication time. ITZ EE was significantly diminished by the exhibition of nano vesicles to sonication for 5 or 15 min (*p* < 0.05). This may be attributed to the perturbation and re-aggregation of vesicles, which coincides with the evasion of a substantial quantity of the drug to the external aqueous milieu. Consequently, the drug is maintained in the aqueous milieu through micellar solubilization rather than being entrapped within ITZ-HA-GLY. This result is acceptable with that of Andersen et al., who reported a decrease in EE as a result of an increase in sonication duration while developing chitosomes and pectosomes for the administration of metronidazole [44].

#### 3.2.2. Determination of Vesicle Size, Polydispersity Index and Zeta Potential of ITZ-HA-GLY

##### VS and PDI Analysis

Small vesicles were ideal for ITZ-HA-GLY preparation because our goal was intratracheal ITZ delivery. The size of ITZ-HA-GLY vesicles was determined using Photon Correlation Spectroscopy, with a range of 187.43 ± 4.49 to 566.54 ± 9.65, as illustrated in Table 2. The surface response curves in Figure 1b illustrates the overall effect of the four independent variables on the VS of the ITZ-HA-GLY vesicles at the median level of the third variable.

The ANOVA test for the detected VS in the ITZ-HA-GLY data suggests that the quadratic model was significant and appropriate for the data. The tailored ITZ-HA-GLY exhibited a small size distribution and remarkable homogeneity, as evidenced by PDI values ranging from 0.10 to 0.34. The quadratic model was used to fit the overall impact of the independent variables on the VS of ITZ-HA-GLY, as indicated by the following Formula (4):

VS = 336.66 − 94.43X_1_ + 48.49X_2_ − 6.74X_3_ − 30.38X_4_ + 27.62X_1_X_2_ + 37.89X_1_X_3_ − 4.77X_1_X_4_ + 21.35X_2_X_3_ − 9.54X_2_X_4_ − 3.05X_3_X_4_ (4)

Augmenting the soybean phosphatidylcholine concentration results in a reduction in vesicle size as it provides a greater amount of soybean phosphatidylcholine component for trapping ITZ and expands the available surface area for trapping. The augmentation of drug encapsulation within vesicles led to enhanced membrane packing and a subsequent reduction in size [45]. Also, the presence of tween 80 content decreases the interfacial tension between 80, the soybean phosphatidylcholine components, and the aqueous component, resulting in a decrease in VS [46].

Conversely, the concentration of HA had a noticeable impact on the VS of ITZ-HA-GLY, as depicted in Figure 1b. The inclusion of HA resulted in an increase in VS, which can be attributed to the adsorption or insertion of anionic hyaluronan molecules onto the surface of cationic ITZ molecules during the creation of ITZ-HA-GLY [47].

Also, Figure 1b demonstrates the impact of sonication duration on the average size of vesicles. The data obtained showed a linear decrease in the average vesicle’s diameter of ITZ-HA-GLY as the sonication time increased. This may come from the exposure of the vesicles to ultrasonic vibrations, causing the dissipation of HA-GLY into smaller sizes. Moreover, the degradation of the polymeric backbone of HA caused a decrease in the rate of decrement in the average VS from 5 or 15 min, which also led to the relaxation of the treated ITZ-HA-GLY structure [48].

##### ZP

The ZP values displayed in Table 2 varied from 25.49 ± 0.83 mV to 43.23 ± 0.43 mV. To prevent misunderstanding, we shall debate about ZP variation in terms of its absolute value, because all the formulations in our investigation exhibited negative Zeta potential values. Since ± 30 mV is considered typical for stability, these values are perfect for the formulation’s stability. The electrostatic repulsion forces resulting from the surface charge overpower the van der Waals attractive forces between 80 them, so facilitating the achievement of stability [49].

The ANOVA test determined that the quadratic model was both statistically significant and appropriate for fitting the observed ZP data. The formula provides the quantitative influence of the three causal factors on the ZP ITZ-HA-GLY, expressed in coded values, as shown in Formula (5):

ZP = 39.00 + 6.75X_1_ − 1.84X_2_ −0.81X_3_ + 3.49X_4_ − 0.22X_1_X_2_ − 0.12X_1_X_3_ + 0.85 X_1_X_4_ + 0.019 X_2_X_3_ − 0.37X_2_X_4_ − 0.21X_3_X_4_ (5)

Soybean phosphatidylcholine concentration has a positive effect on ZP because phosphatidylcholine is a zwitterionic molecule; its head group’s orientation and conformation are not constant and can be affected by binding or adsorption of negatively charged molecules; and HA can create a structured vesicle–polymer system by interacting with the choline groups on both surfaces of the bilayer [50,51].

The results of the ANOVA showed that HA and tween 80 had a substantial impact on the ZP values (*p* < 0.0001). The increased amount of the HA content led to the creation of greater vesicles with reduced zeta potentials, attributable to the negative charge of the HA. The nanovesicles with a low zeta potential have the ability to come together and settle due to the force of electrostatic repulsion, causing the vesicles to enlarge [52]. Wadhwa et al. found evidence that adding HA to chitosan nanoparticles reduced their ZP [53].

In addition, the inclusion of tween 80 in the ITZ-HA-GLY resulted in a reduction in the negative ZP, as seen in Figure 1c. This can be related to the fact that tween 80 investigated tend to be located on the outer layer of the vesicular bilayers because of their hydrophilic nature [54].

According to the ZP concept, the duration of sonication is another influential component that has a substantial impact on ZP. The observed impact can be ascribed to the increase in sonication duration, which enhances the specific surface area of ITZ-HA-GLY, thereby elevating its ZP [55]

#### 3.2.3. Selection of the Optimal Formulation

The objective of optimizing pharmaceutical processing is to deliver a high-quality product with optimal physicochemical attributes by modifying the formulation’s independent variables [56]. Hence, the desirability function was used to pick the optimized dispersion from the constructed 16 ITZ-HA-GLY materials based on the proposed 24 full factorial design. The desirability constraints for optimizing dispersion were examined, with a focus on maximizing EE and ZP while minimizing VS. The total desirability value of 0.79, as shown in Figure 1d, was used to evaluate the success of these restrictions. The ideal composition was created by employing soybean phosphatidylcholine (4%), HA (0.1%), and tween 80 (0.2%) with a sonication time of 15 min. The optimized ITZ-HA-GLY was sonicated for 15 min, resulting in an EE of 73.65 ± 1.76%, VS of 210.23 ± 6.43 nm, and ZP of 41.06 ± 2.62. The values obtained from the optimal formulation closely matched the predicted values, as shown in Table 4. The prediction error ranged from 1.5 to 3.16% for different responses, indicating that the proposed mathematical model accurately predicts the dependent responses. Therefore, this formulation was selected for additional examination.

### 3.3. Morphology of ITZ-HA-GLY

**Figure 2:**
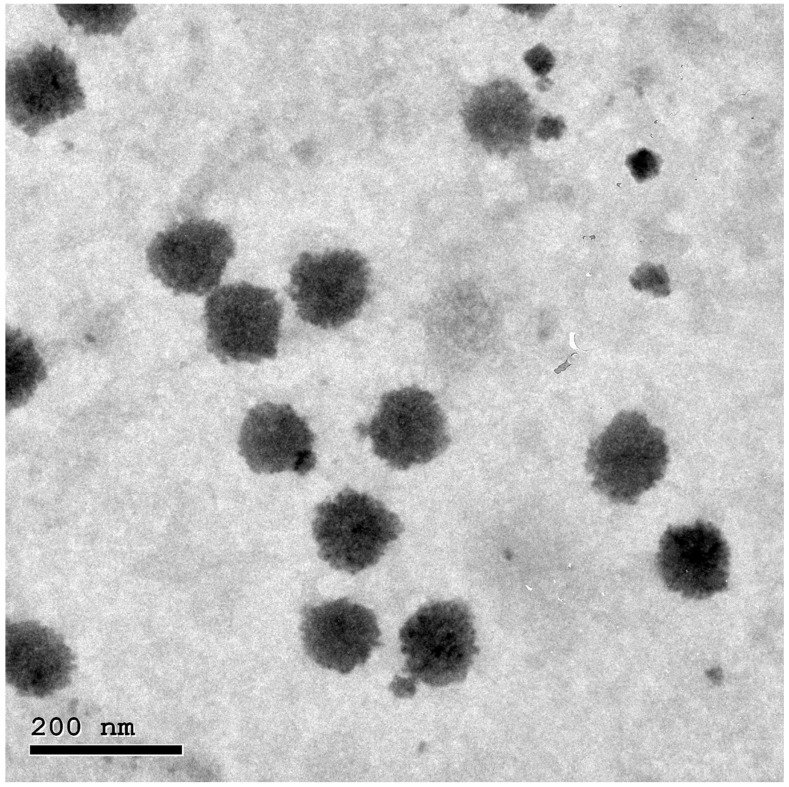
Transmission electron micrograph of the optimal ITZ-HA-GLY formulation.

The morphology of the optimized ITZ-HA-GLY was examined using transmission electron microscopy (TEM) imaging, as illustrated in Figure 2. The vesicles that were observed exhibited a spherical and homogeneous shape, were evenly distributed without any indications of clustering, and exhibited relatively smooth surfaces. The size achieved by transmission electron microscopy (TEM) is smaller than that obtained by dynamic light scattering using a Zetasizer NanoZS (Malvern Instrument) due to the different analysis principles involved in each technique. The resultant size distribution from dynamic light scattering (DLS) represents the average hydrodynamic size of nanoparticles and is often affected by the presence of larger particles, dust, or aggregates [27]. In particular, the nanoparticles that are measured using DLS techniques are in solution and surrounded by nonmoving layers of the medium, which increases their recorded diameter. Microscopic examination using TEM predominantly relies on nanoparticle tracking analysis (NTA), with observations generally performed after the air-drying of nanoparticle droplets on the TEM grid as a conventional method. NTA is a method that employs numerical data to monitor individual nanoparticles (single-particle tracking). The latter can thus yield a precise numerical average dimension with little bias for artifact-free data [27]. As therefore, DLS analysis will produce a larger size than TEM analysis.

### 3.4. In Vitro Drug Release

**Figure 3:**
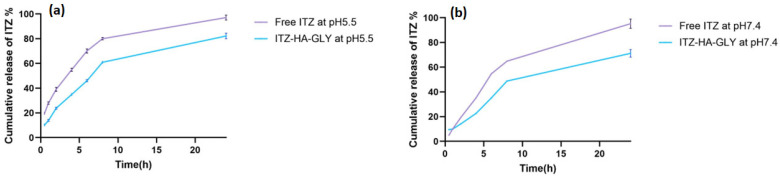
In vitro release profile of ITZ from ITZ suspension and optimized ITZ-HA-GLY formulation at (**a**) pH 5.5 and (**b**) pH 7.4.

GLY in PBS at pH 5.5 and pH 7.4, simulating the acidic endosome and the normal physiological environment, respectively for 24 h, as illustrated in Figure 3. The release investigation demonstrated a notable sustained release pattern for ITZ-HA-GLY in comparison to ITZ suspension. This might be attributed to the prepared ITZ-HA-GLY’s capacity to delay the release of ITZ. At 2 h, there was an initial fast release of ITZ from ITZ-HA-GLY at two different pHs which was significantly higher than the release observed through 24 h.

According to the results, 97.14 ± 2.05% of ITZ was released from ITZ suspension at pH = 5.5, whereas this amount was 95.18 ± 4.8% at pH = 7.4. On the other hand, ITZ exhibited a drug release from ITZ-HA-GLY of approximately 81.19 ± 2.29% and 71.23 ± 3.09% at pH 5.5 and 7.4, respectively. The pH sensitivity of the resultant ITZ-HA-GLY exhibited accelerated drug release at acidic pH levels. The ITZ-HA-GLY released ITZ more rapidly in the acid environment, which is likely a result of the efficient protonation of the ITZ molecule and the ability to evaluate the electrostatic interactions between the positively charged drug and the ionic HA [57]. Zarepour, et al. prepared curcumin-loaded niosome and Rose Bengal enclosed within a chitosan-g-PVCL shell, showing faster release at acidic conditions (pH = 5.5) than at physiological pH conditions (pH = 7.4) [58].

Furthermore, drug release from polymeric nanocarrier systems can occur by diffusion, attrition of the polymer, degradation of the polymer, or desorption on particle surfaces. Additionally, Lipophilic drug is released from ITZ-HA-GLY through diffusion into the aqueous medium, as they pass through the tensioactive interfacial barrier [59].

Various kinetic models were used for the 24 h data of ITZ release as shown in Table 5. The Higuchi diffusion model was fitted to in vitro release finding for ITZ-HA-GLY optimized as indicated by determining coefficient value ITZ-HA-GLY. According to Higuchi’s square root model, several investigations have shown that drug-based vesicular systems provide a controlled release [41,60].

ITZ-HA-GLY formulations have gained prominence in contemporary medical therapies. To attain the appropriate therapy efficacy and safety profiles, it is essential to precisely control the drug release kinetics of the nanoparticles. [61]. In our study, ITZ release kinetics from HA-GLY were investigated by the DD-Solver software program(version 2007). The kinetic modeling of ITZ release from HA-GLY is presented in Table 5. The R^2^ values for the First-order, Higuchi, and Hopfenberg models are lower than those of other models for ITZ-HA-GLY at pH 5.5 and pH 7.4, indicating relatively low values. This indicates that medication release does not conform to these models. Upon examination of the 24-h kinetics results, the values of R^2^, MSC, and AIC were very similar for both the Korsmeyer–Peppas and Peppas–Sahlin models. In other terms, the Korsmeyer–Peppas and Peppas–Sahlin models exhibited a stronger correlation. Drug release from ITZ-HA-GLY has been previously reported to be compatible with numerous models [62]. Some parameters were examined to further elucidate release kinetics. The ‘n’ value is the diffusional exponent indicating the drug release mechanism, especially in the Korsmeyer–Peppas model. The ‘n’ value was found to be 0.572 and 0.663 for ITZ-HA-GLY at pH 5.5 and pH 7.4, respectively, indicating a non-Fickian or anomalous type of transport. The non-Fickian release is described by two mechanisms (a combination of drug diffusion and polymer relaxation). A value of ‘n’ < 0.5 indicates that drug release from the delivery sys-tem corresponds to the Quasi-Fickian diffusion [63]. The alignment of drug release from the ITZ-HA-GLY with the Peppas–Sahlin model suggests non-Fickian diffusion [64]. In conclusion, the release of ITZ from ITZ-HA-GLY is primarily influenced by a mechanism that encompasses both Fickian (pure diffusion phenomenon) and non-Fickian release (resulting from the relaxation of the polymer chain within the networks) [62].

### 3.5. In Vivo Biodistribution Study

To achieve a therapeutic drug level at the site of lung cancer, the maximum tolerable dose is usually given orally to the patient. For ITZ, which is poorly soluble, a high dose either orally or intravenously is necessary, potentially increasing the possibility of detrimental adverse side effects resulting from elevated drug serum concentrations. Targeting the drug directly to the cancer site (i.e., the lung) could reduce the total amount of drug needed and minimize side effects by lowering systemic drug levels. Moreover, targeted therapy may lead to a faster therapeutic response and potentially shorten the overall treatment duration [65].

#### ITZ Biodistribution Studies

To verify the high localization of ITZ in the lungs other than remaining organs and reduce its systematic concentration and consequently reduce its side effects after Intratracheal administration, as shown in Table 6, biodistribution studies of ITZ were conducted from both ITZ-HA-GLY and ITZ suspension in healthy rats.

**Figure 4:**
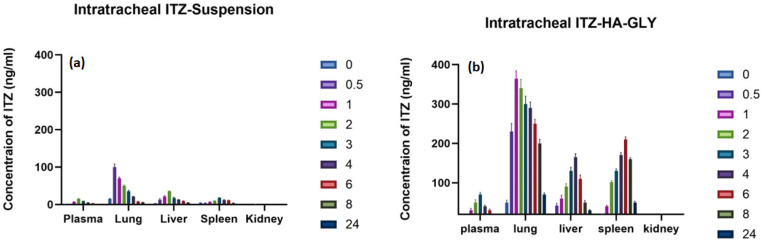
Biodistribution of ITZ in different organs after single administration of (**a**) ITZ-Suspension and (**b**) ITZ-HA-GLY administration (n = 6).

Figure 4 shows ITZ tissue distribution in different organs after intratracheal administration of ITZ-HA-GLY and ITZ suspension. In the spleen, the concentration of ITZ-HA-GLY attained the peak (210 ± 22 ng/mL) after 6 h from drug administration. The splenic ITZ uptake after ITZ suspension was remarkably low throughout the whole experiment period with maximum concentration after 3 h (18 ± 2.5 ng/mL). In the spleen, the highest ITZ uptake found in the ITZ-HA-GLY treatment was 11 times higher than that obtained after ITZ suspension. Upon indicating the uptake level of ITZ by the liver after ITZ-HA-GLY administration, it was found that the maximum concentration (165 ± 12 ng/mL) was reached after 4 h, while for the group treated with ITZ suspension, the maximum concentration (35 ± 3.31 ng/mL) was attained 2 h after the administration. The observed high concentrations that were found in the liver and spleen comparable to the other organs after ITZ-HA-GLY administration is mainly due to two reasons: the first one is the opsonization by phagocytes beside macrophages that are in close contact with the blood [66], and the second reason is that the metabolism of ITZ primarily occurs in the liver through the action of CYP3A4, resulting in the formation of numerous metabolites [67]. However, our findings were much lowered if we compare them to the results previously reported by Elaine P. who developed PLGA-DMSA nanoparticles loaded with ITZ and reported high uptake of this nanoparticle by liver and spleen due to opsonization [34] because of the stealth coat of hyaluronic acid that forms a hydration layer surrounding ITZ-GLY that prevent immune recognition and prolong its life cycle [68].

In the lungs, as revealed in Table 6, the concentration of ITZ from suspension attained the peak (100 ± 15.26 ng/mL) after 30 min from drug administration and then declined quickly with MRT 6 h. In contrast, the concentration of ITZ in ITZ-HA-GLY reached maximum concentration after 1 h (364 ± 22 ng/mL) and declined more slowly with MRT 14 h. The superiority of ITZ-HA-GLY compared to Free ITZ suspension in residing in the lung was due to the addition of hyaluronic acid polymer to glycerosomes. This can be attributed to the polymer’s role that has a critical role in creating a viscous dispersion, which enhances the adhesiveness and deposition of the vesicles in the lung tissue [50].

The levels of ITZ are seen in the kidney after both ITZ-HA-GLY and ITZ suspension administration were below the limit of quantification for ITZ-HA-GLY and ITZ suspension; this may be attributed to the controlled release of ITZ and due to the metabolism of ITZ that mainly takes place inside the liver by several pathways that lead to producing more than 30 undetectable metabolites [34,69].

Throughout the 24 h experiment, the least concentration of ITZ was quantified in the plasma, indicating minimal systemic distribution and reduced accumulation in non-target tissues. As a result, the side effects commonly associated with ITZ in conventional treatments are expected to be diminished [13]. These findings concurred with those presented by Cunha-Azevedo et al. during the preparation of PLGA-DMSA nanoparticles loaded with itraconazole, Mitidieri et al. while increasing gallium availability in the lung using Intratracheal administration, and Manca et al. during nanoincorporation of Curcumin in polymer-glycerosomes [31,33].

### 3.6. In Vitro Cytotoxicity

**Figure 5:**
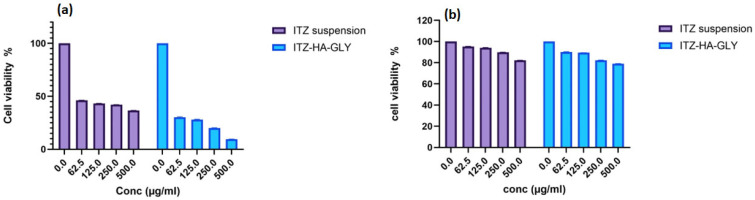
Cell viability of ITZ suspension and ITZ-HA-GLY formulation on (**a**) A549 cancer cell and (**b**) HSF-1 normal cell.

The cytotoxicity of ITZ-HA-GLY produced was evaluated by comparing it with ITZ suspension across a concentration range of 62.50 to 500.00 µg/mL. The A549 cell lines were used, and the cells were subject to the compounds for 24 h at 37 °C. The optimized ITZ-HA-GLY formulation and ITZ suspension exhibited cytotoxic effects that varied depending on the concentration. The cytotoxicity of ITZ-HA-GLY was greater than the activity exhibited by ITZ suspension. The cytotoxicity of ITZ-suspension on lung cell lines was observed to be concentration-dependent, with an IC50 value of 28.14 ± 1.6 µg/mL as shown in Figure 5a. ITZ was found to severely decrease the viability of A549 cells and promote cell death through apoptosis. This was primarily caused by changes in the mitochondria membrane potential, a decrease in Bcl-2 expression, and an increase in caspa-se-3 activity [70].

The optimized ITZ-HA-GLY that was generated substantially decreased the IC50 to 13.03 ± 0.2 µg/mL, as shown in Figure 5. This potentiating effect could be ascribed to the increased permeability into cancer cells and the greater cytotoxic effect on this cell line. The vesicle size was a crucial formulation parameter that influenced cellular absorption efficiency, hence regulating the adhesion strength between 80 nano-vesicles and the cellular surface. Moreover, the augmented permeability and retention would lead to the accumulation of nano-vesicles in tumor tissues [71]. In addition, the incorporation of ITZ into ITZ-HA-GLY significantly enhanced the uptake and activity against a drug-resistant cell line. This increase might be attributed to the bypassing of the efflux P-gp system in these resistant strains as a result of the presence of tween 80 and soybean phosphatidylcholine as excipients in the ITZ-HA-GLY formulation. On the other hand, The carboxyl groups of HA served as active sites that recognized CD44 receptors on tumor cell surfaces, hence facilitating nanoparticle absorption [72,73,74]. Consequently, ITZ-HA-GLY could be more effectively absorbed through receptor-mediated endocytosis, which resulted in a greater amount of ITZ being delivered to cells, thereby enhancing cytotoxicity. These outcomes corroborated the results of Yu-Li Lo et al., who found that the incorporation of lipd significantly influenced the uptake and permeation of epirubicin [75].

#### Cytotoxicity on Normal Cells and Duration of Cytotoxicity Assay

To ensure the biosafety and cytotoxic effect of ITZ suspension and ITZ-HA-GLY on the HSF-1 cell line, which is most prevalent and accessible normal human cell line was utilized, and the produced formulations were evaluated within the same dosage range as the A549 cell line, revealing low and non-significant cytotoxicity (*p* ≤ 0.05), as shown in Figure 5b. ITZ suspension and ITZ-HA-GLY showed less toxicity to human normal cells as compared to A549 cell lines. The possible explanation of these effects is due to the normal cell line having low anabolic demands respire predominantly by glycolytic means with a lower glucose/nutrient intake, and low proliferation rate compared to cancerous cells, which makes them not exposed to ITZ suspension and ITZ-HA-GLY [76].

## 4. Conclusions

The present in vitro and in vivo findings of this study underline the promising properties of HA-GLY as appropriate carriers for ITZ delivery to the lung. The association of sodium hyaluronate and glycerol to the liposomes led to obtaining a suitable ITZ formulation that was not toxic in vitro with low IC50 and had great anticancer activity. Additionally, this formulation was highly biodistributed and provided a high ITZ pulmonary deposition via successfully targeting CDD4 receptors that overexpressed in cancer cells and enhanced EPR. Our results indicate that an appropriate integration of intratracheal administration with nanotechnology could significantly enhance the local pharmacological activity of ITZ, decrease the frequency of daily dosages, mitigate potential adverse effects, and improve therapeutic effectiveness.

## Figures and Tables

**Table 1:** Variables and their corresponding levels in the employed 2^4^ full factorial design for ITZ-HA-GLY.

Variable	Design Level	
	Low (−1)	High (+1)
Independent variables		
A = Soybean phosphatidylcholine concentration (%)	2	4
B = HA concentration (%)	0.1	0.2
C = Tween 80 concentration (%)	0.1	0.2
D = Sonication time (min)	5	15
Dependent variables	Constraints	
Y1 = EE (%)	Maximize	
Y2 = VS (nm)	Minimize	
Y3 = ZP (mW)	Maximize	

HA: hyaluronic acid; EE: entrapment efficiency; VS: vesicle size; ZP: zeta potential.

**Table 2:** The 2^4^ full factorial design and the observed responses of ITZ-HA-GLY.

Formulation	Independent Variables				Dependent Variables			PDI
	X1	X2	X3	X4	Y1: EE	Y2: VS	Y3: ZP	
1	2	0.2	0.2	15	70.25 ± 0.31	429.63 ± 6.5	27.74 ± 0.78	0.23
2	2	0.1	0.1	15	63.46 ± 0.62	465.36 ± 9.14	31.93 ± 0.54	0.19
3	4	0.2	0.2	15	79.07 ± 0.61	484.87 ± 7.07	39.16 ± 0.30	0.21
4	2	0.2	0.2	5	77.92 ± 0.53	475.65 ± 12.0	25.63 ± 0.83	0.31
5	4	0.1	0.2	15	75.32 ± 0.40	187.43 ± 4.49	42.52 ± 0.25	0.34
6	2	0.1	0.2	5	72.58 ± 0.34	420.12 ± 5.96	26.46 ± 0.66	0.18
7	2	0.1	0.2	15	66.45 ± 0.42	381.43 ± 9.89	30.26 ± 0.45	0.11
8	4	0.2	0.2	5	83.97 ± 1.23	495.34 ± 9.03	33.59 ± 0.53	0.13
9	4	0.1	0.2	5	80.07 ± 0.53	230.54 ± 8.48	36.53 ± 0.44	0.19
10	2	0.2	0.1	15	69.35 ± 0.31	520.34 ± 10.0	28.51 ± 0.57	0.15
11	2	0.1	0.1	5	74.61 ± 1.15	491.02 ± 12.4	26.79 ± 0.68	0.21
12	4	0.2	0.1	15	72.65 ± 0.20	285.43 ± 7.16	40.76 ± 0.41	0.12
13	2	0.2	0.1	5	73.78 ± 0.14	566.54 ± 9.65	25.49 ± 0.83	0.17
14	4	0.1	0.1	15	70.94 ± 0.95	201.78 ± 8.86	43.23 ± 0.43	0.15
15	4	0.1	0.1	5	78.45 ± 0.71	225.45 ± 6.03	37.59 ± 0.75	0.16
16	4	0.2	0.1	5	79.84 ± 0.52	335.65 ± 7.37	34.83 ± 0.79	0.10

Data represent mean values (n = 3) ± S.D.

**Table 3:** Analysis of variance of calculated model for measured response.

Parameters	DF	SS	MS	F	*p*-Value
EE%					
Regression	10	438.18	43.82	20.27	0.002
Residual	5	10.81	2.92	1.35	-
Total	15	448.98	-	-	-
Vesicle size					
Regression	10	2.26 × 10^5^	22,639.95	10.93	0.0083
Residual	5	10,360.01	2072.002	-	-
Total	15	2.37 × 10^5^		-	-
Zeta potential					
Regression	10	577.02	57.7	178.28	0.0001
Residual	5	1.62	0.32	-	-
Total	15	578.64	-	-	-

DF: degrees of freedom; SS: sums of squared error; MS: mean squared error (MS = SS/DF); F: Fisher’s ratio (F = MS Regression/MS Residual).

**Table 4:** Composition, actual, and predicted responses of the optimal ITZ-HA-GLY.

Factor	Optimal Value	Response Variable	Actual Value	Predicted Value	% Prediction Error ^a^
A: Soybean phosphatidylcholine (%)	4	EE %	73.65 ± 1.76	74.77	−1.5
B: HA (%)	0.1	VS nm	210.23 ± 6.43	204.57	2.65
C: Tween 80 (%)	0.2	ZP mV	41.06 ± 2.62	42.36	−3.16
D: Sonication time (min)	15	-	-	-	-

EE: entrapment efficiency; VS: vesicle size; ZP: zeta potential. ^a^ Calculated as [Actual-predicted/Actual] × 100.

**Table 5:** Release kinetics from the optimized ITZ-HA-GLY at pH 7.4 and 5.5.

Model and Equation	Evaluation Criteria					
	ITZ-HA-GLY at pH 5.5			ITZ-HA-GLY at pH 7.4		
	R^2^	AIC	MSC	R^2^	AIC	MSC
First-order F = 100 [1 − Exp (−k_1_ × t)]	0.9706	35.1519	3.2404	0.9535	36.7450	2.7817
Higuchi F = k_H_ × t^0.5^	0.9785	32.9635	3.5530	0.9591	35.8460	2.9101
Korsmeyer-Peppas F = k_KP_ × t^n^	0.9820	33.7057	3.4470	0.9742	34.6096	3.0868
Hopfenberg F = 100 × [1 − (1 − k_HB_ × t)^n^]	0.9706	37.1573	2.9539	0.9534	38.7493	2.4954
Peppas-Sahlin F = k_1_ × t^m^ + k_2_ × t^2m^	0.9934	28.7143	4.1600	0.9830	33.7061	3.2159

In each models, F represent is the fraction (%) of drug released in time t, k_1_: first-order release constant, k_H_: Higuchi release constant, k_KP_: release constant incorporating structural and geometric characteristics of the drug-dosage form, n: the diffusional exponent indicating the drug-release mechanism, k_HB_: Hopfenberg release constant, m: the diffusional exponent for a device of any geometric shape which inhibits controlled release. AIC: The Akaike information criterion, MSC: model selection criterion, and R^2^: correlation coefficient.

**Table 6:** Lung Deposition Pharmacokinetic Parameters for ITZ-HA-GLY and ITZ suspension.

Formulation	C_max_ (ng/mL)	T_max_ (h)	T_1/2_ (h)	AUC_(0–24)_ (µgh/mL)	MRT (h)
ITZ suspension	100	0.5	4	186	6
ITZ-HA-GLY	364	1	10	4335	14

## Data Availability

The original contributions presented in the study are included in the article, further inquiries can be directed to the corresponding authors.
